# Development of the Novel FLOX Score to Assess Risk of Intubation in Children With Immunocompromising Conditions Receiving High Flow Nasal Cannula

**DOI:** 10.1002/ppul.71814

**Published:** 2026-08-31

**Authors:** Michelle A. Mai, Colin M. Rogerson, Courtney M. Rowan

**Affiliations:** ^1^ Indiana University School of Medicine Indianapolis Indiana USA; ^2^ Department of Pediatrics, Division of Critical Care Indiana University School of Medicine Indianapolis Indiana USA; ^3^ Riley Hospital for Children at IU Health Indianapolis Indiana USA; ^4^ Regenstrief Center for Biomedical Informatics Indianapolis Indiana USA

**Keywords:** cancer, critical care, Immunocompromised host, Noninvasive ventilation, pediatrics, respiratory distress syndrome, respiratory insufficiency

## Abstract

**Background:**

Heated, humidified, high‐flow nasal cannula (HFNC) before intubation has been associated with increased mortality in children with immunocompromising conditions (ICC).

**Objectives:**

We sought to describe HFNC failure (intubation) rate and aimed to develop a novel score associated with HFNC failure.

**Methods:**

This is a single center, retrospective cohort. Patients (aged 0–26 years) with ICCs who used HFNC between 2021 and 2023 were included.

**Results:**

Forty‐four (47.3%) of 93 included subjects failed HFNC. PICU mortality was 14% (*n* = 13). The median duration of HFNC was 18.7 h (IQR: 6.0, 40.4). We developed the novel FLOX ((flow in Liters/minute)/weight (kg)*FiO_2_) score. Median FLOX score was higher with HFNC failure at initiation (*p* = 0.04), 2 h (*p* = 0.03), 4 h (*p* = 0.05), and 6 h (*p* = 0.05). Adjusting for acuity, the odds of HFNC failure with a FLOX score (≥0.20) were as follows: Initiation OR = 2.5 (95%CI: 1.03, 5.9), *p* = 0.042); 2 h of HFNC, OR = 4.3 ((95%CI: 1.7, 10.9), *p* = 0.002); 4 h of HFNC, OR = 6.5 (95%CI: 2.5, 16.8), (*p* < 0.001); and 6 h of HFNC, OR = 4.9 (95%CI: 1.9, 12.7), (*p* = 0.001).

**Conclusion:**

In children with ICCs, almost half fail HFNC. Mortality was high in those who fail HFNC. The FLOX score, specifically a FLOX ≥ 0.20 is associated with increased odds of failing HFNC in our ICC cohort.

## 1 Introduction

Children with immunocompromising conditions (ICCs) have a higher risk of mortality from acute hypoxic respiratory failure than the general pediatric population [1]. This risk increases further when invasive mechanical ventilation is required. Therefore, noninvasive respiratory support strategies to prevent intubation, such as heated, humidified, high flow nasal cannula (HFNC), in this population are of high interest. Data surrounding noninvasive respiratory support in adults with ICC are mixed. While some studies suggest that HFNC may decrease rates of intubation [2, 3], others, including a large, randomized controlled trial, suggest no significant differences in intubation rates and mortality with HFNC when compared to standard O_2_ therapy [4, 5, 6]. Data on HFNC use in children is lacking. One registry study found that HFNC exposure prior to intubation was common in children, including those with ICC. In this study, HFNC exposure prior to intubation was associated with an increase in mortality, but only in children with ICC [7]. The reasons underlying the increased risk of HFNC failure and mortality in this population remain poorly understood and likely reflect a complex interplay of underlying disease, immune dysfunction, and high rates of organ dysfunction [1]. However, given the high mortality associated with invasive mechanical ventilation and concerns that delayed recognition of HFNC failure may contribute to adverse outcomes, there remains an urgent need to identify children at highest risk for failure while mechanistic studies continue to evolve.

HFNC is frequently used to prevent intubation, by improving inspiratory demand, increasing functional residual capacity, and washing out pharyngeal dead space [8]. However, preventing intubation with HFNC use in children with ICC has not been established. Additionally, limited data suggest that exposure to HFNC before intubation is associated with increased mortality in children with ICC [7]. It is well‐known that children with ICC are at high risk for progressive and severe acute hypoxic respiratory failure, but we do not understand how the use of HFNC impacts this severity. It is possible that HFNC failure in this population may be associated with increased mortality from severe pediatric acute respiratory distress syndrome (PARDS). HFNC criteria are included in the at risk for and possible PARDS definition [9]. However, these definitions have not been well‐explored in those with ICC receiving HFNC. In the context of the high rate of PARDS in this population, risk scores that incorporate measures of delivered FiO_2_ may be important for early identification of patients unlikely to respond to HFNC. The objective of this study was to evaluate the use of HFNC in children with ICC and associated clinical outcomes. Further, we aimed to develop a novel metric of HFNC respiratory support and determine its association with HFNC failure in a cohort of children with ICC.

## 2 Materials and Methods

We conducted a single center, retrospective cohort study using all consecutive patients with ICC, aged 0–26 years, at Riley Hospital for Children who received HFNC from January 2021 to December 2023. Indiana University Institutional Review Board approval (IRB# 23402) and a waiver of informed consent due to the use of existing, de‐identified data was obtained on May 29, 2024. Procedures were followed in accordance with the ethical standards of the responsible committees on human experimentation and with the Helsinki Declaration of 1975. ICCs were defined as active oncology diagnosis undergoing treatment, post‐hematopoietic cell or solid organ transplant within the last year, primary or acquired immunodeficiency, or therapy with an immunocompromising agent, such as systemic corticosteroids, chemotherapy, biologic immunomodulators, and transplant‐related immunosuppressive therapies. Patients were excluded from the study if they only received HFNC after intubation, or if they had care limitations (i.e., do not intubate/resuscitate) within the first 2 days of HFNC therapy. There were 116 subjects initially identified as potentially having an ICC and who received HFNC alone or HFNC prior to intubation. Sixteen were excluded due to not having an active ICC upon manual chart review and an additional seven were excluded due to care limitations. At our institution Optiflow is used to deliver HFNC and there is no protocol for HFNC initiation. While some providers will aim for 1–2 L/Kg of flow in smaller children and 20 L of flow for larger children, this is not standardized. Implementation of HFNC settings is heavily provider dependent.

Patients were initially identified using our center's Virtual Pediatric Systems (VPS LLC) database by querying diagnostic categories of oncologic, immunologic, hematologic, and transplant status and the use of ICD10 codes. Inclusion and exclusion criteria were then confirmed by manual review of the electronic medical record. Data collected included demographics, comorbidities, ICC characteristics, cause of respiratory failure, Pediatric risk of mortality score (PRISM) [10], radiographic findings, laboratory values, infectious data, use of vasoactive agents, and clinical outcomes. Vital signs and respiratory parameters were collected at HFNC initiation (*n* = 93), and 2 h (*n* = 84), 4 h (*n* = 77), and 6 h (*n* = 70) following HFNC initiation. These time points were chosen a priori as we believe them to be clinically meaningful in failure prediction. Cause of respiratory failure was ascertained by review of physician notes in medical records. The primary outcome was HFNC failure defined as needing intubation any time after HFNC initiation. Additional outcomes included mortality, PARDS as defined by PALICC‐2 [9], duration of respiratory support, and length of stay.

### 2.1 Definitions and FLOX Score Development

Applying PALICC‐2 criteria, we knew that many children would meet the threshold for “at‐risk for PARDS” defined as receiving HFNC at 1.5 L/kg or ≥30 L of flow. We initially sought to determine the rate of Possible PARDS. PALICC‐2 does not include HFNC as part of the definition for PARDS. However, it does include HFNC in the possible PARDS definition. Possible PARDS is diagnosed in children receiving ≥ 1.5 L/kg or ≥ 30 L of HFNC and having a PaO_2_/FiO_2_ ratio ≤300 or an SpO_2_/FiO_2_ ratio ≤ 250 [11]. However, in our cohort, we lacked PaO_2_ data and had frequent time points with a SpO_2_ greater than 97%. Therefore, we were not able to accurately determine the PaO_2_/FiO_2_ or the SpO_2_/FiO_2_ [12] for the majority of those on HFNC, limiting our ability to calculate the number with possible PARDS. This also limited calculation and evaluation of the pediatric ROX Index [13], which includes SpO_2_/FiO_2_ in the calculation. In our unit, it is very uncommon to place arterial lines or obtain arterial blood gases in children that are not intubated. However, SpO_2_/FiO_2_ requires saturations to be <98% for the calculation to be valid [14]. This requires attention to weaning FiO_2_ so that target saturations can be obtained. While our unit typically aims for SpO_2_ above 90% and weans FiO_2_ for saturations in the high 90 s, in times of high acuity and volume of patients, weaning FiO_2_ does not always happen efficiently. Further, if the child is having frequent desaturation, the beside providers may be hesitant to wean. These intermittent desaturations, even if they are frequent, may not be documented well in the electronic medical record. Lastly, there are limitations of using SpO_2_ in general, particularly in low perfusions states or patients with darker skin tone [12].

Therefore, to overcome these limitations of SpO_2_ and to better understand the impact of degree of HFNC support we developed and tested the FLOX (FLow OXygen) score association with HFNC failure. This score is calculated with the following equation: [(Flow/Kg)*FiO_2_]. We developed this score to assess the degree of HFNC support characterized by flow. Previous data has demonstrated that increasing flow rates lead to improved work of breathing [15, 16]. We then incorporated the amount of FiO_2_ support as a surrogate to assess the degree of hypoxemia. This score was calculated at initiation of HFNC, and 2, 4, and 6 h following HFNC initiation, or until the patient failed HFNC if that occurred first. The vast majority (>75%) received HFNC for ≥ 6 h.

### 2.2 Statistical Methods

Medians and interquartile ranges were used for continuous variables. Categorical variables were presented using frequencies and percentages of the cohort. Wilcoxon rank sum test was used to compare continuous variables. Chi‐squared or Fishers exact test were used to compare categorical variables. Univariate and multivariable logistic regression was used to assess association with outcomes. Confounding variables entered into the model (PRISM score and cause of respiratory failure) were chosen a priori due to the clinical concern to be associated with outcome. After constructing a receiver operating characteristic curve, Liu's index was used to determine cutpoint for the FLOX score. Liu's index identifies the optimal cutpoint by maximizing the product of sensitivity and specificity [16]. Areas under the curve for FLOX as a continuous variable are provided in Table S1. The optimal cutpoint identified by Liu's index was 0.20. Therefore, a FLOX score ≥ 0.20 was considered high for binary analysis. A *p*‐value < 0.05 was considered statistically significant. Stata SE version 18.0 was used for the analysis.

## 3 Results

Of the 93 patients included in the study cohort, 47.3% (*n* = 44) failed HFNC. The median age of the entire cohort was 9.0 (IQR: 4.0, 15.0) years and 34.3% were female. There were no demographic or characteristic differences found between those with HFNC failure compared to those without HFNC failure (Table 1).

**Table 1:** Patient characteristics of the cohort.

Patient characteristics	HFNC^a^ success *n* = 49	HFNC failure *n* = 44	*p* value
Age in years (median, IQR)	9 (4,16)	8.5 (2,14)	0.27
Female sex	17 (35%)	15 (34%)	0.95
Weight (kg) (median, IQR)	36.3 (18.1, 71)	29.6 (10.7, 53.2)	0.07
Race			0.23
White	43 (88%)	36 (82%)	
Black	2 (4%)	6 (14%)	
Asian	2 (4%)	0 (0%)	
Unknown	2 (4%)	2 (5%)	
Hispanic	7 (14%)	2 (5%)	0.11
Route to intensive care unit admission			0.57
Outside hospital	4 (8%)	7 (16%)	
Emergency department	11 (22%)	7 (16%)	
General care floor	31 (63%)	27 (61%)	
Operating/procedural suite, post anesthesia recovery unit	2 (4%)	3 (7%)	
Other	1 (2%)	0 (0%)	
Immunocompromising condition			0.42
Malignancy	30 (31%)	20 (46%)	
Post‐hematopoietic cell transplant	8 (16%)	12 (27%)	
Post‐solid organ transplant	6 (12%)	3 (7%)	
Bone marrow failure syndromes	2 (4%)	3 (7%)	
Primary/Acquired immunodeficiency	1 (2%)	3 (7%)	
Other	2 (4%)	0	
PRISM^b^ Score (median, IQR)	5 (0, 12)	7 (2,10)	0.52
Cause of Respiratory Failure			0.26
Pulmonary/direct lung injury	34 (69%)	35 (80%)	
Extrapulmonary/indirect lung injury	15 (31%)	9 (20%)	

a HFNC = High flow nasal canula.

^b^ PRISM = Pediatric risk of mortality.

### 3.1 At‐Risk for PARDS, Possible PARDS, and Respiratory Parameters Associated With HFNC Failure

We first investigated the association of at‐risk for PARDS criteria with HFNC failure. Thirty‐nine percent (*n* = 36) of children with ICC met at‐risk for PARDS criteria within the first 6 h of HFNC therapy. Being at risk for PARDS at any point during these first 6 h was associated with HFNC failure [OR: 3.7 (95% CI: 1.5, 8.9), *p* = 0.004]. Details of those at risk for PARDS at individual time points of HFNC and the association with HFNC failure can be found in Table S2.

**Figure 1:**
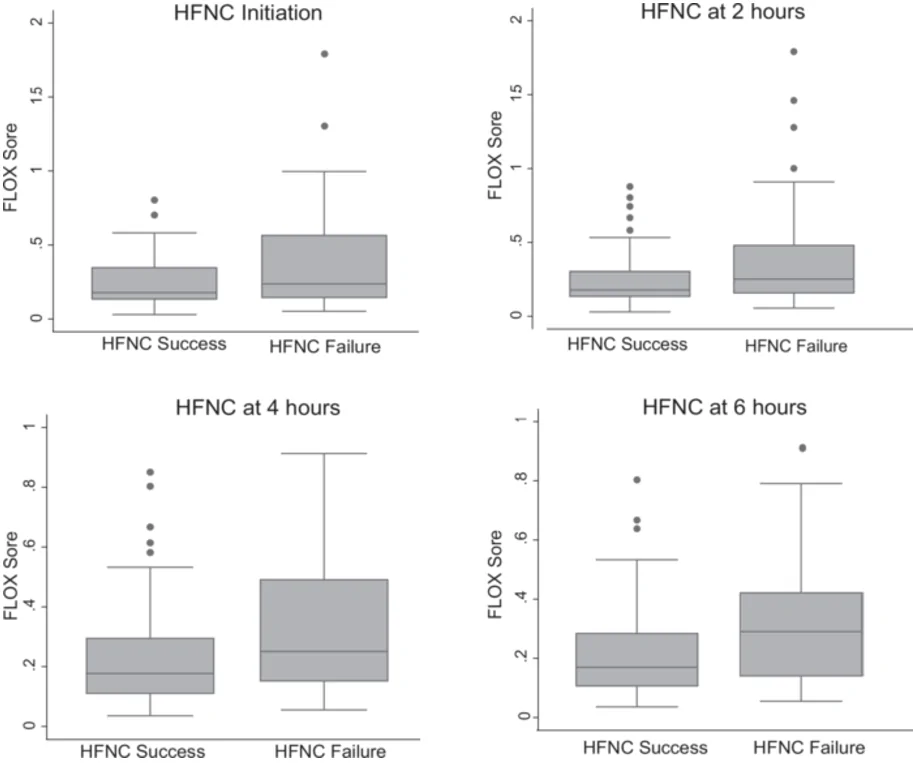
Box plots comparing the median and distribution of the FLOX score between those that failed HFNC and those who were successfully treated. FLOX scores were evaluated at HFNC initiation and at 2, 4, and 6 h following HFNC initiation. FLOX = liter flow/minute/kg*FiO_2_.

**Figure 2:**
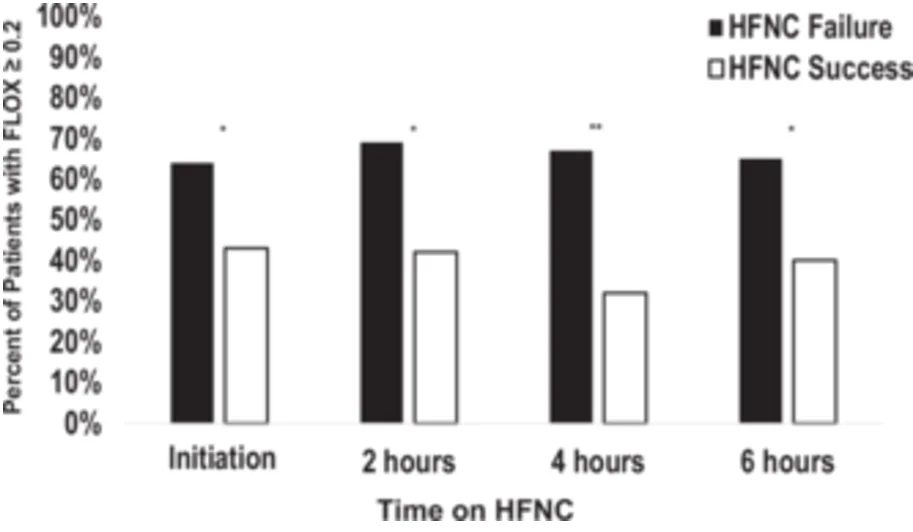
This figure demonstrates the percent of patients in each group with a FLOX score ≥ 0.20 at high flow nasal canula (HFNC) initiation and at 2, 4, and 6 h post high flow nasal cannula initiation. Those who failed HFNC are illustrated with the black bars. Those who were successfully treated with HFNC are illustrated with white bars.

Those who failed HFNC had higher median FLOX at 2 h (*p* = 0.03), and statistically trending to be higher at 4 h (*p* = 0.05) and 6 h (*p* = 0.05) (Figure 1). To make the model more clinically applicable, a FLOX score value of 0.20 was used as a cutpoint. This cutpoint was derived using Liu's index. Using this cutpoint a FLOX score ≥ 0.20 was associated with HFNC failure. A significantly greater proportion of those who failed HFNC had a FLOX score of ≥ 0.20 at HFNC initiation (*p* = 0.045), at 2 h of HFNC (*p* = 0.012), at 4 h of HFNC use (*p* = 0.003), and at 6 h of HFNC use (*p* = 0.039) (Figure 2). Those with a FLOX score ≥ 0.20 had an unadjusted, statistically significant increased odds in HFNC at all four time points measured (Table S3). The increased odds of failure for those with a high FLOX score persisted after adjusting for admission PRISM score and cause of respiratory failure (Table 2). Models adjusted for age and weight with similar results can be found in the supplement (Tables S4 and S5). We also investigated if improvement in FLOX score over time was associated with HFNC success, but this was not statistically significant (Table S6).

**Table 2:** Adjusted odds ratio for high flow nasal canula failure for children with immunocompromising conditions and a FLOX^a^ score ≥0.20.

Time on HFNC^b^	Adjusted odds ratio^c^ (95% CI)	*p*‐value	AUC (95%CI)
**Initial**	2.5 (95% CI: 1.03, 5.9)	0.042	0.65 (0.54, 0.76)
**2 h**	4.3 (95% CI: 1.7, 10.9)	0.002	0.71 (0.61, 0.82)
**4 h**	6.5 (95% CI: 2.5, 16.8)	< 0.001	0.75 (0.65, 0.85)
**6 h**	4.9 (95% CI: 1.9, 12.7)	0.001	0.72 (0.62, 0.82)

^a^ FLOX = Flow rate/Kg*FiO_2._

^b^ HFNC = High flow nasal canula.

^c^ Adjusted for PRISM (Pediatric Risk of Mortality) score at ICU admission and cause of respiratory failure.

### 3.2 Other Clinical Outcomes

The median duration of HFNC use was 18.7 h (IQR: 6.0, 40.4). As expected, those successfully treated with HFNC had a longer duration of HFNC (*p* < 0.001) and shorter length of PICU stay (*p* < 0.001) (Table 3). There was no difference in rates of escalation to BIPAP. Thirteen patients (14%) died in the PICU. Three of these patients were never intubated after having care limitations instituted later in the PICU course. Of the patients that were intubated 10/44 (23%) died in the PICU. Out of those who were intubated, 28/44 (64%) had PARDS upon intubation. Of note, all 10 who died had PARDS at intubation. Four patients who survived the ICU course died prior to hospital discharge.

**Table 3:** Clinical outcomes compared between those who were successful managed with HFNC^a^ therapy and those who failed HFNC therapy.

Outcome	Entire cohort *n* = 93	HFNC success *n* = 49	HFNC failure *n* = 43	*p* value
Duration of HFNC (hours)	18.7 (6.0, 40.4)	33.8 (14.9, 61.2)	8.2 (3.6, 20.6)	< 0.001
Escalation to BIPAP^b^ *n* (%)	25 (27%)	14 (29%)	11 (25%)	0.70
Length of PICU^c^ stay (days)	6.0 (3.0, 15.0)	4.0 (3.0, 7.0)	11.5 (6.0, 20.0)	< 0.001
PICU mortality *n* (%)	13 (14%)	3 (6%)	10 (23%)	0.02

*Note:* Continuous variables are presented as medians with (interquartile ranges).

^a^ HFNC = High flow nasal cannula.

^b^ BIPAP = Bilevel positive airway pressure.

^c^ PICU = Pediatric intensive care unit.

## 4 Discussion

In this cohort of 93 children with ICCs who received HFNC between 2021 and 2023, HFNC failure rate was high with almost half the cohort going on to be intubated. Mortality was high in those who failed HFNC. Both meeting at‐risk for PARDS criteria and a FLOX score ≥ 0.20 were associated with HFNC failure. Additionally, there was a high rate of PARDS on intubation in the failure cohort with all patients that died meeting criteria for PARDS. This suggests that oxygenation deficits and PARDS may be key drivers for both HFNC failure and mortality in those with ICC.

In our cohort of those with ICC, almost half of the patients failed HFNC. The high rate of HFNC failure is similar to published studies of other noninvasive respiratory support. In a study of 153 children post‐allogenic hematopoietic cell transplant who used noninvasive positive pressure ventilation, over 60% of patients failed their noninvasive support and needed to be intubated [17]. This failure rate is also similar to what has been reported in studies of adults with ICC [4, 6]. The consistent high failure rate reported in previous pediatric and adult literature demonstrates that children with ICC receiving HFNC are a high‐risk, vulnerable population that deserve close monitoring for progressive respiratory failure.

The respiratory rate‐oxygenation (ROX) and ROX heart rate (ROX‐HR) have been shown in general critically ill children to be associated with HFNC failure [18]. As we found in our cohort, these scores can also be limited by the validity of the SpO_2_/FiO_2_ ratio at all pulse oximetry readings [12]. In order for the SpO_2_/FiO_2_ ratio to be valid, SpO_2_ must be ≤ 97% which can be affected by the attention to respiratory support adjustments at the bedside. The ROX and ROX‐HR scores are also more complex in children where there is variation in age related vital sign norms. The FLOX score is simple, can quickly be calculated at the bedside and is less dependent on adjusting for age‐related variability. It is important to note that due to the lack of data, a direct comparison of FLOX to ROX and ROX‐HR could not be done. However, scores developed in the general pediatric population often do not apply to children with ICC [19]. The reason for this is likely multifactorial. There are well documented studies demonstrating ICCs to be a risk factor for the development of PARDS [20]. Given the risk for PARDS development, we originally sought to examine the association of at risk for PARDS and possible PARDS with HFNC [9]. While we were able to identify those meeting at‐risk for PARDS criteria to be at higher odds to develop HFNC, we were not able to assess the risk with possible PARDS. Possible PARDS criteria sets an oxygenation threshold of PaO_2_/FiO_2_ ≤ 300 or SpO_2_/FiO_2_ ≤ 250 [11]. However, within our cohort, we frequently could not use these formulas due to the lack of arterial blood gas information and often patients having documented oxygenation saturations > 97%. Due to the retrospective nature of our study, it is difficult to determine if the frequent documentation of high pulse oximetry saturations is secondary to a failure to wean FiO_2_ or due to choosing to document vitals at the best moment. Regardless, due to this limitation, we developed the FLOX score to assess the degree of respiratory support incorporating both the flow rate and FiO_2_. We found the FLOX score to be associated with HFNC failure. The FLOX score was higher in those who failed HFNC at initiation, 2, 4, and 6 h of HFNC support. We were also able to identify a clinically applicable cutpoint in this cohort. FLOX score > 0.20 is associated with increased odds of failing HFNC. The clinical implication of this value is that it can be calculated early in treatment, even at initiation of HFNC use, to determine the likelihood of failure. This can improve early identification of those who will fail to help avoid delayed intubation and to aid in prognostic counseling to families.

To support the importance of oxygenation deficits as a driver of HFNC, there was a high rate of PARDS upon intubation in this cohort. Of those who failed HFNC, 64% met PARDS criteria on intubation. Additionally, we found that of those who failed HFNC and died in the PICU, all (*n* = 10) met PARDS criteria on intubation. This is not surprising given the previous studies demonstrating high rates of PARDS mortality in children with ICC [1, 11, 20]. While it seems that oxygenation failure may be a driver of both HFNC failure and related mortality, the question remains if clinicians can modify this risk. ICC is also a known risk factor for noninvasive respiratory support failure in PARDS [21]. HFNC exposure prior to intubation is associated with increased respiratory failure mortality, but only in children with ICC [7]. The reasons behind this are unclear but one postulated mechanism is the potential for delayed intubation leading to worse outcomes. Therefore, it is imperative that HFNC failure be recognized early. Our novel FLOX score may help identify those patients within the first few hours of HFNC therapy.

Given our study's retrospective nature, there are limitations due to the available data. It is difficult to retrospectively understand the indications behind medical decisions, including intubation, and other factors of patient care. The cause of HFNC failure is very challenging to determine retrospectively. While 64% of those that failed had PARDS upon intubation, leading us to speculate that refractory hypoxemia was a key driver of HFNC failure, it is unclear to what degree other factors such as hypercarbia, severe work of breathing, shock, altered mental status or multi‐organ dysfunction may have contributed. This type of study can lead to an increase in unknown confounding variables. This study was a single center study; therefore, the sample size was small. This could introduce limitations to the power, reproducibility, and applicability of the results to a larger population. The FLOX score was significant across age and weight. This could be perhaps that these factors are less important in the ICC population, which tends to be older. However, our sample size was also small, prohibiting and in‐depth look at various age and weight categories to confirm if the FLOX score is truly age dependent. A high FLOX score of > 0.20 was associated with HFNC at all four timepoints studied. While optimal cutpoints at individual times may have higher predictive value, there is simplicity of use to have the same cutpoint at various times. This may improve clinical implementation. The FLOX score was created through our research at a single center; therefore, further research incorporating data from other centers should be done to validate this score in children with ICC. This is especially important with our modest AUCs that may be improved with future research.

In conclusion, we found that HFNC failure rate is high in those with ICCs. Further, mortality is high in those who fail HFNC. We demonstrated that those meeting at‐risk for PARDS criteria were at higher odds to fail HFNC. We also discovered that the FLOX score ≥ 0.20 is associated with HFNC in our single center cohort of children with ICC. The rate of Pediatric Acute Respiratory Distress Syndrome on intubation was high in those who failed HFNC. These results need to be explored in an external cohort for further validation.

## Author Contributions

**Michelle A. Mai:** conceptualization, writing – original draft, methodology, writing – review and editing, data curation, funding acquisition. **Colin M. Rogerson:** methodology, writing – review and editing, conceptualization. **Courtney M. Rowan:** conceptualization, funding acquisition, methodology, writing – review and editing, formal analysis, software, supervision, data curation, writing – original draft.

## Conflicts of Interest

The authors declare no conflicts of interest.

## Supporting information


Supporting File 1

## Data Availability

The data that support the findings of this study are available from the corresponding author upon reasonable request.
