# Supplementary material for: Development of the Novel FLOX Score to Assess Risk of Intubation in Children With Immunocompromising Conditions Receiving High Flow Nasal Cannula
Source: Pediatr Pulmonol. 2026 Aug 31;61(9):e71814. doi: 10.1002/ppul.71814 (PMC13527891; doi:10.1002/ppul.71814)
Supplement: Supplementary file 1 [file PPUL-61-0-s001.docx]

Supplemental materials for:

***Development of the Novel FLOX Score to assess risk of intubation in children with immunocompromising conditions receiving high flow nasal cannula***

Authors:

Michelle Mai^1^, Colin M. Rogerson, MD, MPH^2,3,4^, Courtney Rowan, MD, MS^2,3^

Table S1: Areas under the curve for FLOX as a continuous variable at various time points.

| FLOX score | AUC (95% Confidence interval) |
| --- | --- |
| - Initial | 0.63 (0.51, 0.74) |
| - 2 hours | 0.64 (0.52, 0.76) |
| - 4 hours | 0.63 (0.50, 0.77) |
| - 6 hours | 0.63 (0.50, 0.78) |

Table S2. At Risk for PARDS comparison assess by HFNC success and failure over the first 6 hours of HFNC use

| **Timing of HFNC** | **HFNC success** | **HFNC failure** | **p-value** | **Unadjusted OR (95%CI)** |
| --- | --- | --- | --- | --- |
| **Initiation** | 7 (14%) | 5 (11%) | 0.675 | 0.8 (0.2, 2.6) |
| **2 hours** | 8 (16%) | 15 (34%) | 0.047 | 2.7 (1.0, 7.1) |
| **4 hours** | 8 (16%) | 20 (45%) | 0.002 | 4.3 (1.6,11.2) |
| **6 hours** | 11 (22%) | 24 (55%) | 0.001 | 4.1 (1.7, 10.2) |

Table S3. Unadjusted odds ratio for HFNC failure for children with ICC and a FLOX score ≥ 0.20 at different times of HFNC use

|  | **Odds Ratio (95% CI)** | **p-value** | **Model AUC** |
| --- | --- | --- | --- |
| **Initial** | 2.3 (95% CI: 1.01, 5.4) | 0.047 | 0.60 |
| **2 hours** | 3.1 (95% CI: 1.3, 7.9) | 0.013 | 0.66 |
| **4 hours** | 4.3 (95% CI: 1.6, 11.3) | 0.004 | 0.71 |
| **6 hours** | 2.8 (95% CI: 1.04, 7.7) | 0.042 | 0.67 |

Table S4: Age adjusted Odds ratio for HFNC failure for children with ICC and FLOX score ≥ 0.20 at different times of HFNC use.

|  | **Odds Ratio (95% CI)** | **p-value** |
| --- | --- | --- |
| **Initial** | 2.6 (95% CI: 1.0, 6.8) | 0.054 |
| **2 hours** | 5.1 (95% CI: 1.8, 14.5) | 0.002 |
| **4 hours** | 10.5 (95% CI: 3.2, 34.3) | <0.001 |
| **6 hours** | 5.8 (95% CI: 2.1, 16.6) | 0.001 |

Model was adjusted for PRISM, cause of respiratory failure, and age. Of note, age was not significantly associated in this model with HFNC failure at any of the time points.

Table S5: Weight adjusted Odds ratio for HFNC failure for children with ICC and FLOX score ≥ 0.20 at different times of HFNC use.

|  | **Odds Ratio (95% CI)** | **p-value** |
| --- | --- | --- |
| **Initial** | 2.0 (95% CI: 0.7, 5.6) | 0.180 |
| **2 hours** | 4.2 (95% CI: 1.4, 12.4) | 0.010 |
| **4 hours** | 7.4 (95% CI: 2.4, 22.6) | <0.001 |
| **6 hours** | 4.8 (95% CI: 1.6, 14.3) | 0.004 |

Model was adjusted for PRISM, cause of respiratory failure, and age. Of note, weight was not significantly associated in this model with HFNC failure at any of the time points.

Table S6: Improving FLOX scores were not associated with HFNC success/failure

|  | Odds ratio (95% confidence interval) | p value |
| --- | --- | --- |
| Compared to initial FLOX | | |
| - 2 hours | 2.0 (95% CI: 0.7, 5.7) | 0.196 |
| - 4 hours | 0.9 (95% CI; 0.3, 2.1) | 0.723 |
| - 6 hours | 0.5 (95% CI: 0.2, 1.2) | 0.109 |
| Compared to 2 hour FLOX | | |
| - 4 hours | 0.5 (95% CI: 0.2, 1.5) | 0.217 |
| - 6 hours | 0.5 (95% CI: 0.2, 1.3) | 0.144 |

*Improvement in FLOX was categorized as a binary variable with “improvement” being defined as having a lower FLOX score of any degree when compared to the preceding FLOX score.
